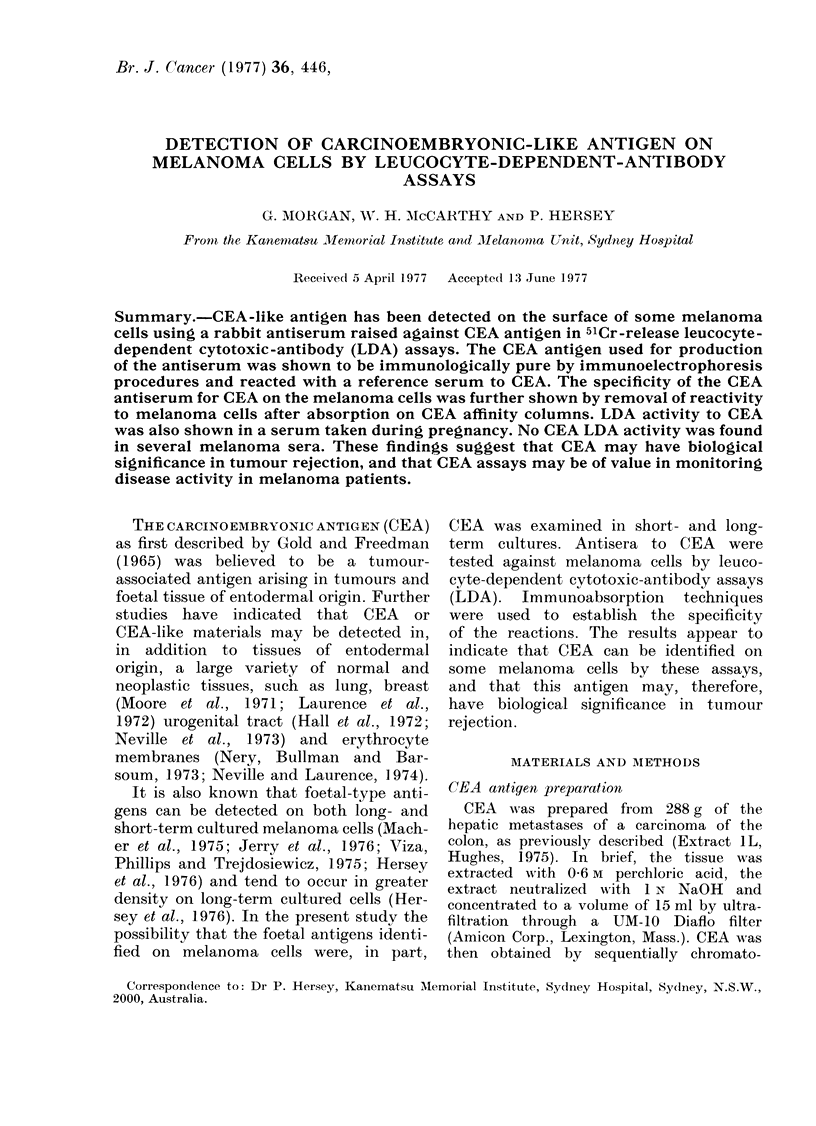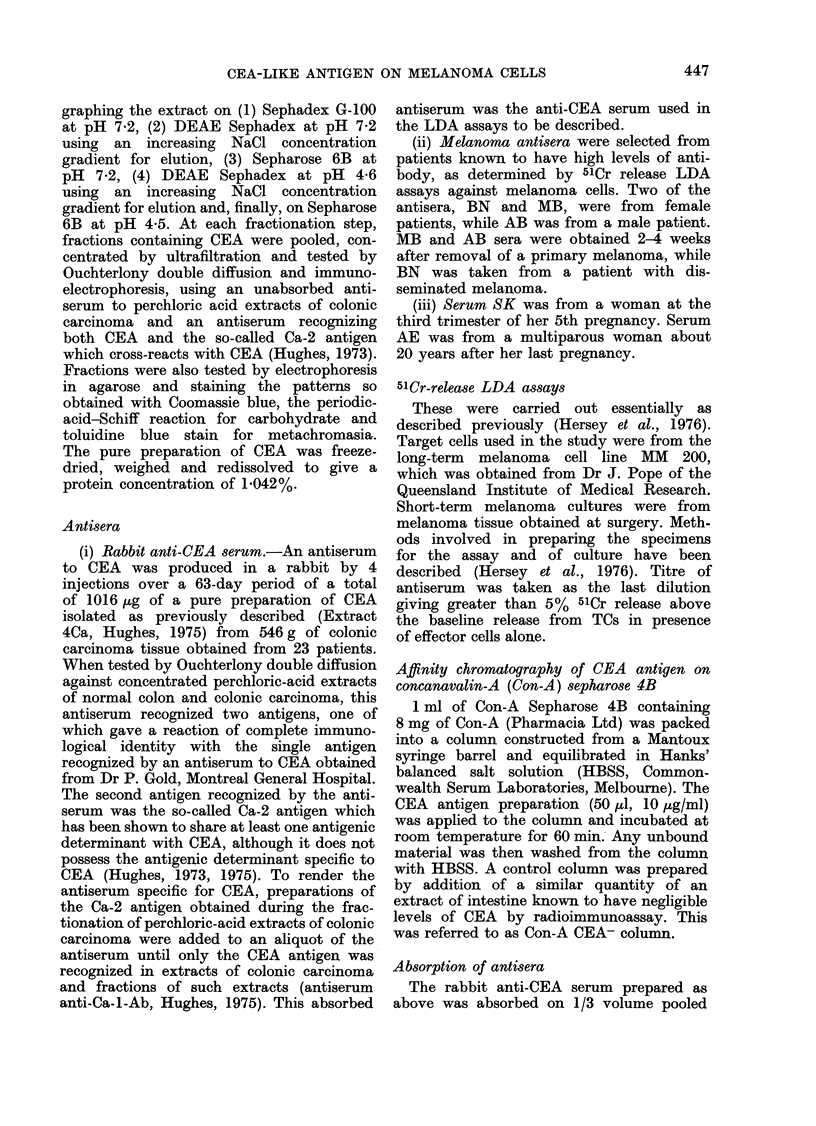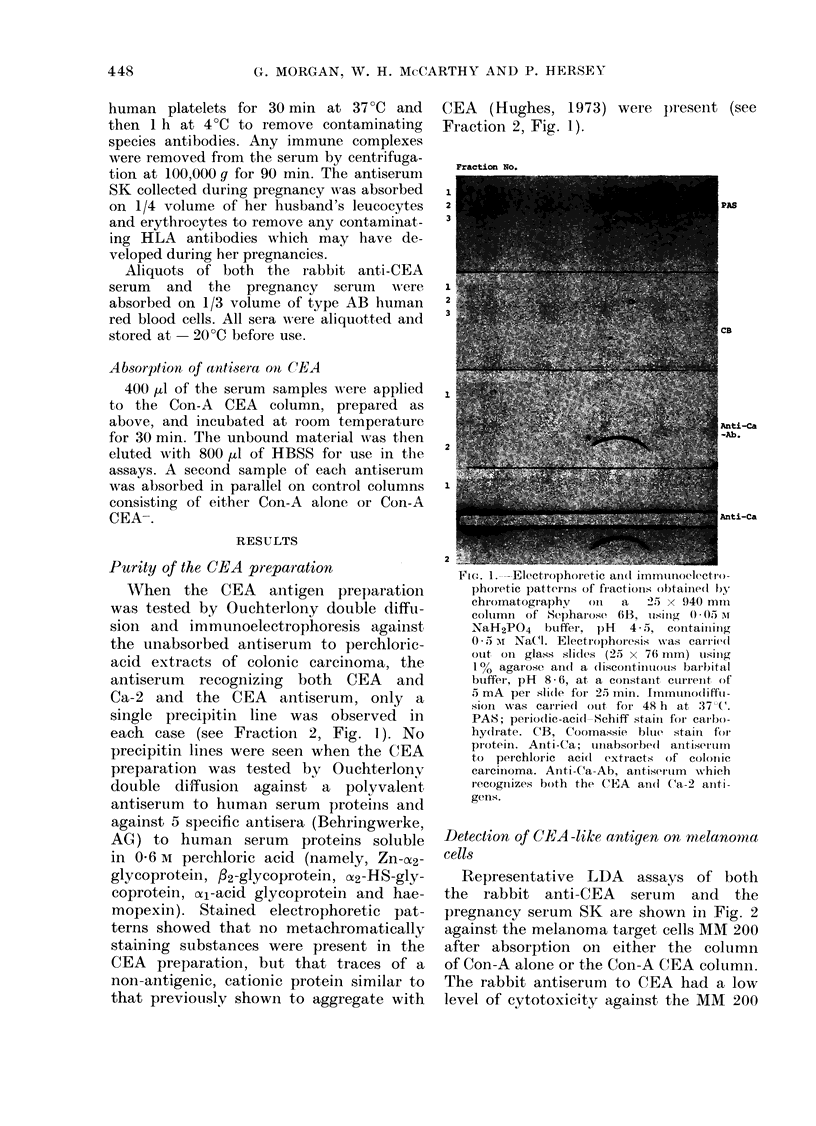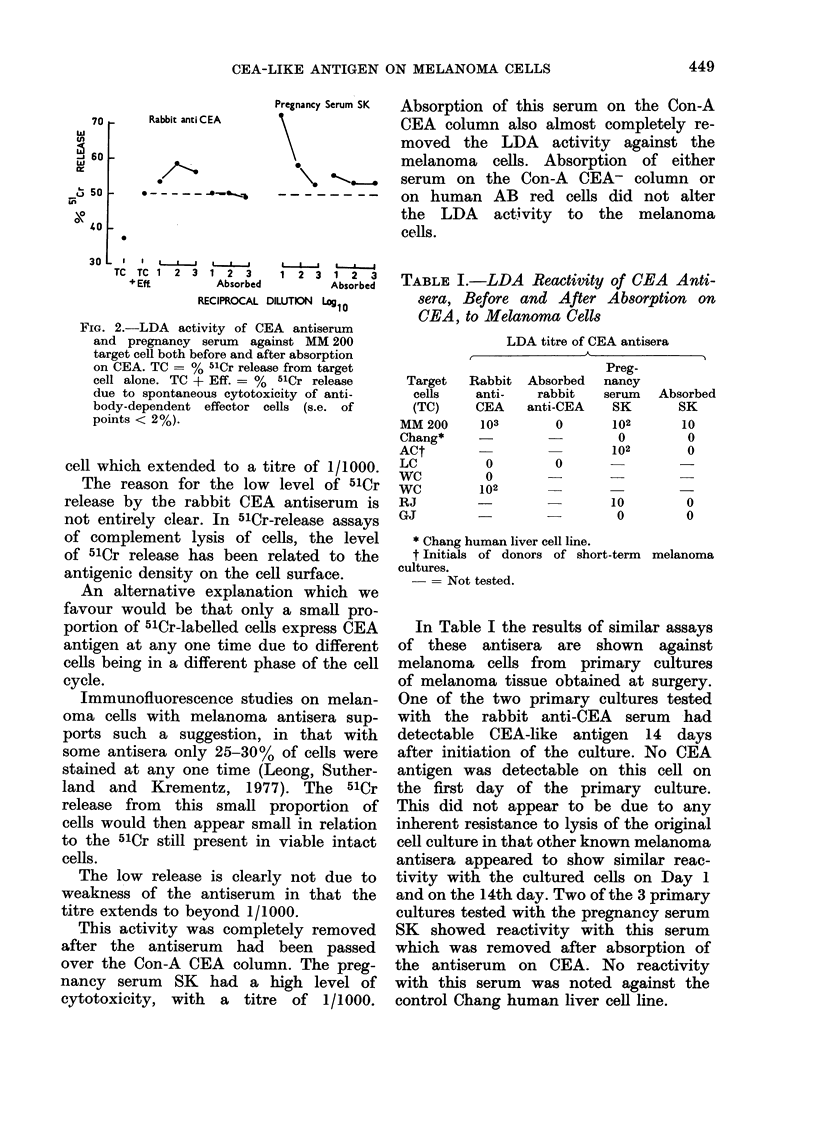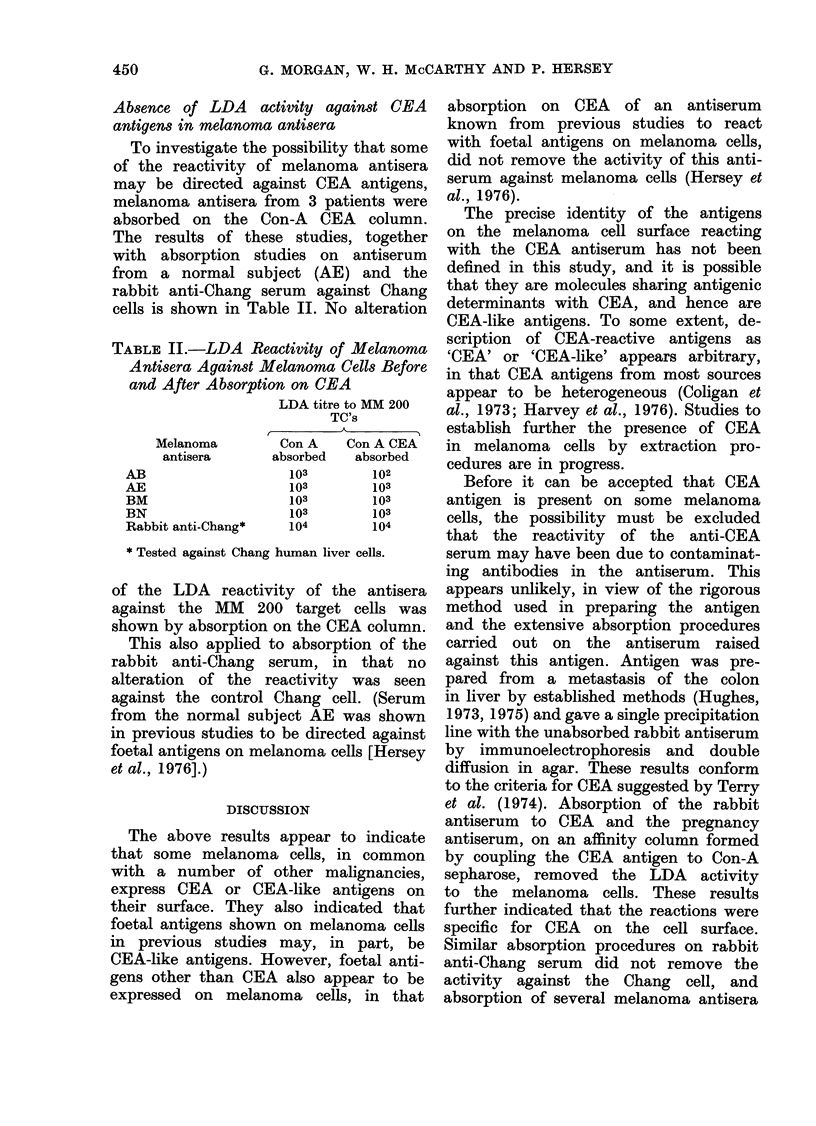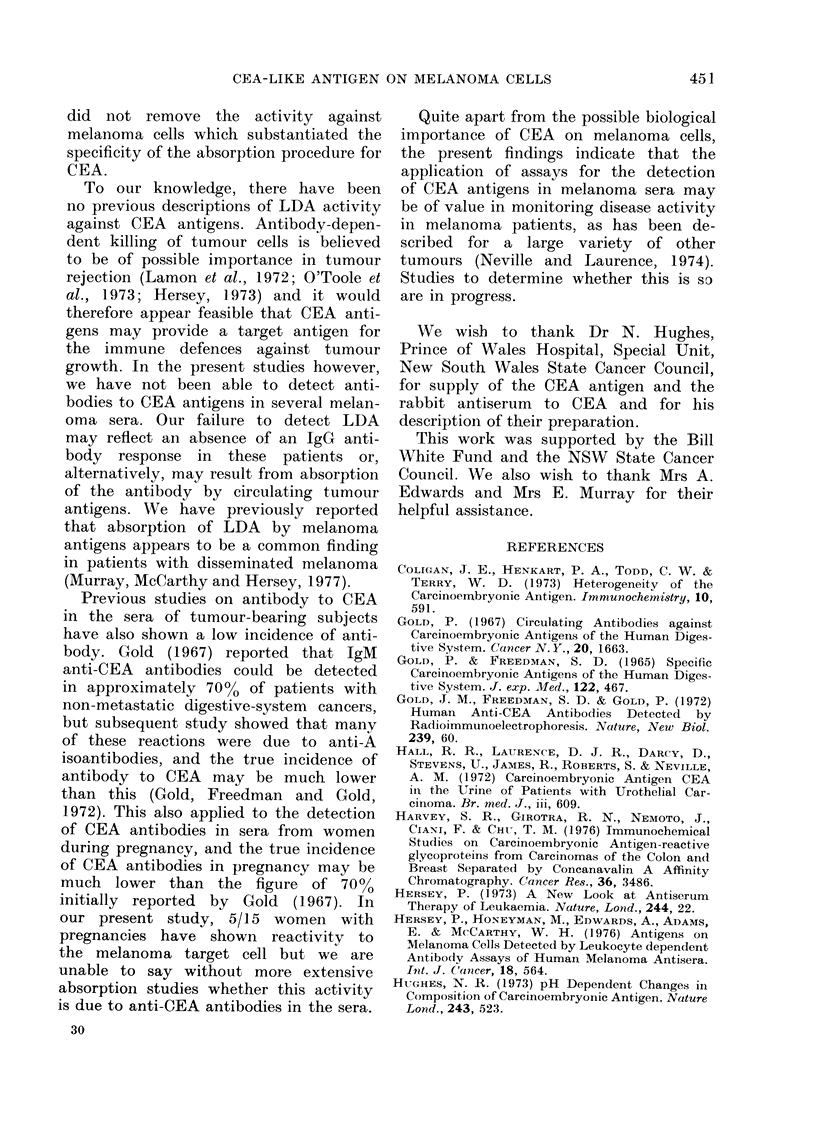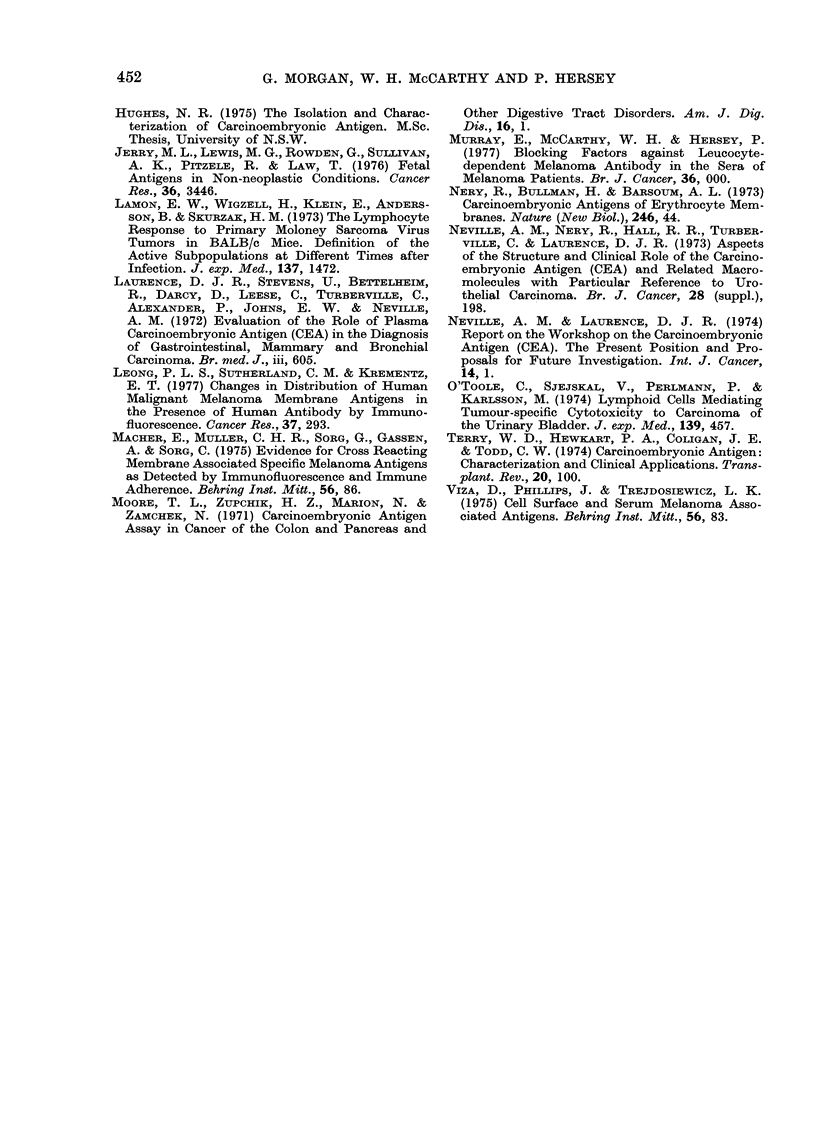# Detection of carcinoembryonic-like antigen on melanoma cells by leucocyte-dependent-antibody assays.

**DOI:** 10.1038/bjc.1977.213

**Published:** 1977-10

**Authors:** G. Morgan, W. H. McCarthy, P. Hersey

## Abstract

**Images:**


Br. J. Cancer (1977) **36**, 446,

### DETECTION OF CARCINOEMBRYONIC-LIKE ANTIGEN ON MELANOMA CELLS BY LEUCOCYTE-DEPENDENT-ANTIBODY ASSAYS

G. MORGAN, W. H. McCARTHY AND P. HERSEY

*From the Kanematsu Memorial Institute and Melanoma Unit, Sydney Hospital*

Received 5 April 1977 Accepted 13 June 1977

**Summary.—CEA-like antigen has been detected on the surface of some melanoma cells using a rabbit antiserum raised against CEA antigen in $^{51}$Cr-release leucocyte-dependent cytotoxic-antibody (LDA) assays. The CEA antigen used for production of the antiserum was shown to be immunologically pure by immunoelectrophoresis procedures and reacted with a reference serum to CEA. The specificity of the CEA antiserum for CEA on the melanoma cells was further shown by removal of reactivity to melanoma cells after absorption on CEA affinity columns. LDA activity to CEA was also shown in a serum taken during pregnancy. No CEA LDA activity was found in several melanoma sera. These findings suggest that CEA may have biological significance in tumour rejection, and that CEA assays may be of value in monitoring disease activity in melanoma patients.**

THE CARCINOEMBRYONIC ANTIGEN (CEA) as first described by Gold and Freedman (1965) was believed to be a tumour-associated antigen arising in tumours and foetal tissue of entodermal origin. Further studies have indicated that CEA or CEA-like materials may be detected in, in addition to tissues of entodermal origin, a large variety of normal and neoplastic tissues, such as lung, breast (Moore *et al.*, 1971; Laurence *et al.*, 1972) urogenital tract (Hall *et al.*, 1972; Neville *et al.*, 1973) and erythrocyte membranes (Nery, Bullman and Barsoum, 1973; Neville and Laurence, 1974).

It is also known that foetal-type antigens can be detected on both long- and short-term cultured melanoma cells (Macher *et al.*, 1975; Jerry *et al.*, 1976; Viza, Phillips and Trejdosiewicz, 1975; Hersey *et al.*, 1976) and tend to occur in greater density on long-term cultured cells (Hersey *et al.*, 1976). In the present study the possibility that the foetal antigens identified on melanoma cells were, in part, CEA was examined in short- and long-term cultures. Antisera to CEA were tested against melanoma cells by leucocyte-dependent cytotoxic-antibody assays (LDA). Immunoabsorption techniques were used to establish the specificity of the reactions. The results appear to indicate that CEA can be identified on some melanoma cells by these assays, and that this antigen may, therefore, have biological significance in tumour rejection.

### MATERIALS AND METHODS

### *CEA antigen preparation*

CEA was prepared from 288 g of the hepatic metastases of a carcinoma of the colon, as previously described (Extract 1L, Hughes, 1975). In brief, the tissue was extracted with 0·6 M perchloric acid, the extract neutralized with 1 N NaOH and concentrated to a volume of 15 ml by ultrafiltration through a UM-10 Diaflo filter (Amicon Corp., Lexington, Mass.). CEA was then obtained by sequentially chromato-

Correspondence to: Dr P. Hersey, Kanematsu Memorial Institute, Sydney Hospital, Sydney, N.S.W., 2000, Australia.

graphing the extract on (1) Sephadex G-100 at pH 7·2, (2) DEAE Sephadex at pH 7·2 using an increasing NaCl concentration gradient for elution, (3) Sepharose 6B at pH 7·2, (4) DEAE Sephadex at pH 4·6 using an increasing NaCl concentration gradient for elution and, finally, on Sepharose 6B at pH 4·5. At each fractionation step, fractions containing CEA were pooled, concentrated by ultrafiltration and tested by Ouchterlony double diffusion and immunoelectrophoresis, using an unabsorbed antiserum to perchloric acid extracts of colonic carcinoma and an antiserum recognizing both CEA and the so-called Ca-2 antigen which cross-reacts with CEA (Hughes, 1973). Fractions were also tested by electrophoresis in agarose and staining the patterns so obtained with Coomassie blue, the periodic-acid–Schiff reaction for carbohydrate and toluidine blue stain for metachromasia. The pure preparation of CEA was freeze-dried, weighed and redissolved to give a protein concentration of 1·042%.

*Antisera*

(i) *Rabbit anti-CEA serum.*—An antiserum to CEA was produced in a rabbit by 4 injections over a 63-day period of a total of 1016 μg of a pure preparation of CEA isolated as previously described (Extract 4Ca, Hughes, 1975) from 546 g of colonic carcinoma tissue obtained from 23 patients. When tested by Ouchterlony double diffusion against concentrated perchloric-acid extracts of normal colon and colonic carcinoma, this antiserum recognized two antigens, one of which gave a reaction of complete immunological identity with the single antigen recognized by an antiserum to CEA obtained from Dr P. Gold, Montreal General Hospital. The second antigen recognized by the antiserum was the so-called Ca-2 antigen which has been shown to share at least one antigenic determinant with CEA, although it does not possess the antigenic determinant specific to CEA (Hughes, 1973, 1975). To render the antiserum specific for CEA, preparations of the Ca-2 antigen obtained during the fractionation of perchloric-acid extracts of colonic carcinoma were added to an aliquot of the antiserum until only the CEA antigen was recognized in extracts of colonic carcinoma and fractions of such extracts (antiserum anti-Ca-1-Ab, Hughes, 1975). This absorbed antiserum was the anti-CEA serum used in the LDA assays to be described.

(ii) *Melanoma antisera* were selected from patients known to have high levels of antibody, as determined by $^{51}$Cr release LDA assays against melanoma cells. Two of the antisera, BN and MB, were from female patients, while AB was from a male patient. MB and AB sera were obtained 2–4 weeks after removal of a primary melanoma, while BN was taken from a patient with disseminated melanoma.

(iii) *Serum SK* was from a woman at the third trimester of her 5th pregnancy. Serum AE was from a multiparous woman about 20 years after her last pregnancy.

*$^{51}$Cr-release LDA assays*

These were carried out essentially as described previously (Hersey *et al.*, 1976). Target cells used in the study were from the long-term melanoma cell line MM 200, which was obtained from Dr J. Pope of the Queensland Institute of Medical Research. Short-term melanoma cultures were from melanoma tissue obtained at surgery. Methods involved in preparing the specimens for the assay and of culture have been described (Hersey *et al.*, 1976). Titre of antiserum was taken as the last dilution giving greater than 5% $^{51}$Cr release above the baseline release from TCs in presence of effector cells alone.

*Affinity chromatography of CEA antigen on concanavalin-A (Con-A) sepharose 4B*

1 ml of Con-A Sepharose 4B containing 8 mg of Con-A (Pharmacia Ltd) was packed into a column constructed from a Mantoux syringe barrel and equilibrated in Hanks' balanced salt solution (HBSS, Commonwealth Serum Laboratories, Melbourne). The CEA antigen preparation (50 μl, 10 μg/ml) was applied to the column and incubated at room temperature for 60 min. Any unbound material was then washed from the column with HBSS. A control column was prepared by addition of a similar quantity of an extract of intestine known to have negligible levels of CEA by radioimmunoassay. This was referred to as Con-A CEA$^-$ column.

*Absorption of antisera*

The rabbit anti-CEA serum prepared as above was absorbed on 1/3 volume pooled human platelets for 30 min at 37°C and then 1 h at 4°C to remove contaminating species antibodies. Any immune complexes were removed from the serum by centrifugation at 100,000 *g* for 90 min. The antiserum SK collected during pregnancy was absorbed on 1/4 volume of her husband's leucocytes and erythrocytes to remove any contaminating HLA antibodies which may have developed during her pregnancies.

Aliquots of both the rabbit anti-CEA serum and the pregnancy serum were absorbed on 1/3 volume of type AB human red blood cells. All sera were aliquotted and stored at − 20°C before use.

*Absorption of antisera on CEA*

400 μl of the serum samples were applied to the Con-A CEA column, prepared as above, and incubated at room temperature for 30 min. The unbound material was then eluted with 800 μl of HBSS for use in the assays. A second sample of each antiserum was absorbed in parallel on control columns consisting of either Con-A alone or Con-A $CEA^-$.

RESULTS

*Purity of the CEA preparation*

When the CEA antigen preparation was tested by Ouchterlony double diffusion and immunoelectrophoresis against the unabsorbed antiserum to perchloric-acid extracts of colonic carcinoma, the antiserum recognizing both CEA and Ca-2 and the CEA antiserum, only a single precipitin line was observed in each case (see Fraction 2, Fig. 1). No precipitin lines were seen when the CEA preparation was tested by Ouchterlony double diffusion against a polyvalent antiserum to human serum proteins and against 5 specific antisera (Behringwerke, AG) to human serum proteins soluble in 0·6 M perchloric acid (namely, Zn-$\alpha_2$-glycoprotein, $\beta_2$-glycoprotein, $\alpha_2$-HS-glycoprotein, $\alpha_1$-acid glycoprotein and haemopexin). Stained electrophoretic patterns showed that no metachromatically staining substances were present in the CEA preparation, but that traces of a non-antigenic, cationic protein similar to that previously shown to aggregate with CEA (Hughes, 1973) were present (see Fraction 2, Fig. 1).

FIG. 1.—Electrophoretic and immunoelectrophoretic patterns of fractions obtained by chromatography on a 25 × 940 mm column of Sepharose 6B, using 0·05 M $NaH_2PO_4$ buffer, pH 4·5, containing 0·5 M NaCl. Electrophoresis was carried out on glass slides (25 × 76 mm) using 1% agarose and a discontinuous barbital buffer, pH 8·6, at a constant current of 5 mA per slide for 25 min. Immunodiffusion was carried out for 48 h at 37°C. PAS; periodic-acid–Schiff stain for carbohydrate. CB, Coomassie blue stain for protein. Anti-Ca; unabsorbed antiserum to perchloric acid extracts of colonic carcinoma. Anti-Ca-Ab, antiserum which recognizes both the CEA and Ca-2 antigens.

*Detection of CEA-like antigen on melanoma cells*

Representative LDA assays of both the rabbit anti-CEA serum and the pregnancy serum SK are shown in Fig. 2 against the melanoma target cells MM 200 after absorption on either the column of Con-A alone or the Con-A CEA column. The rabbit antiserum to CEA had a low level of cytotoxicity against the MM 200

FIG. 2.—LDA activity of CEA antiserum and pregnancy serum against MM 200 target cell both before and after absorption on CEA. TC = % $^{51}$Cr release from target cell alone. TC + Eff. = % $^{51}$Cr release due to spontaneous cytotoxicity of antibody-dependent effector cells (s.e. of points < 2%).

cell which extended to a titre of 1/1000.

The reason for the low level of $^{51}$Cr release by the rabbit CEA antiserum is not entirely clear. In $^{51}$Cr-release assays of complement lysis of cells, the level of $^{51}$Cr release has been related to the antigenic density on the cell surface.

An alternative explanation which we favour would be that only a small proportion of $^{51}$Cr-labelled cells express CEA antigen at any one time due to different cells being in a different phase of the cell cycle.

Immunofluorescence studies on melanoma cells with melanoma antisera supports such a suggestion, in that with some antisera only 25–30% of cells were stained at any one time (Leong, Sutherland and Krementz, 1977). The $^{51}$Cr release from this small proportion of cells would then appear small in relation to the $^{51}$Cr still present in viable intact cells.

The low release is clearly not due to weakness of the antiserum in that the titre extends to beyond 1/1000.

This activity was completely removed after the antiserum had been passed over the Con-A CEA column. The pregnancy serum SK had a high level of cytotoxicity, with a titre of 1/1000. Absorption of this serum on the Con-A CEA column also almost completely removed the LDA activity against the melanoma cells. Absorption of either serum on the Con-A CEA$^-$ column or on human AB red cells did not alter the LDA activity to the melanoma cells.

TABLE I.—*LDA Reactivity of CEA Antisera, Before and After Absorption on CEA, to Melanoma Cells*

| | LDA titre of CEA antisera | | | |
|---|---|---|---|---|
| Target cells (TC) | Rabbit anti-CEA | Absorbed rabbit anti-CEA | Pregnancy serum SK | Absorbed SK |
| MM 200 | $10^3$ | 0 | $10^2$ | 10 |
| Chang* | — | — | 0 | 0 |
| AC† | — | — | $10^2$ | 0 |
| LC | 0 | 0 | — | — |
| WC | 0 | — | — | — |
| WC | $10^2$ | — | — | — |
| RJ | — | — | 10 | 0 |
| GJ | — | — | 0 | 0 |

* Chang human liver cell line.
† Initials of donors of short-term melanoma cultures.
— = Not tested.

In Table I the results of similar assays of these antisera are shown against melanoma cells from primary cultures of melanoma tissue obtained at surgery. One of the two primary cultures tested with the rabbit anti-CEA serum had detectable CEA-like antigen 14 days after initiation of the culture. No CEA antigen was detectable on this cell on the first day of the primary culture. This did not appear to be due to any inherent resistance to lysis of the original cell culture in that other known melanoma antisera appeared to show similar reactivity with the cultured cells on Day 1 and on the 14th day. Two of the 3 primary cultures tested with the pregnancy serum SK showed reactivity with this serum which was removed after absorption of the antiserum on CEA. No reactivity with this serum was noted against the control Chang human liver cell line.

*Absence of LDA activity against CEA antigens in melanoma antisera*

To investigate the possibility that some of the reactivity of melanoma antisera may be directed against CEA antigens, melanoma antisera from 3 patients were absorbed on the Con-A CEA column. The results of these studies, together with absorption studies on antiserum from a normal subject (AE) and the rabbit anti-Chang serum against Chang cells is shown in Table II. No alteration

TABLE II.—*LDA Reactivity of Melanoma Antisera Against Melanoma Cells Before and After Absorption on CEA*

| Melanoma antisera | LDA titre to MM 200 TC's: Con A absorbed | Con A CEA absorbed |
|---|---|---|
| AB | $10^3$ | $10^2$ |
| AE | $10^3$ | $10^3$ |
| BM | $10^3$ | $10^3$ |
| BN | $10^3$ | $10^3$ |
| Rabbit anti-Chang* | $10^4$ | $10^4$ |

* Tested against Chang human liver cells.

of the LDA reactivity of the antisera against the MM 200 target cells was shown by absorption on the CEA column.

This also applied to absorption of the rabbit anti-Chang serum, in that no alteration of the reactivity was seen against the control Chang cell. (Serum from the normal subject AE was shown in previous studies to be directed against foetal antigens on melanoma cells [Hersey *et al.*, 1976].)

### DISCUSSION

The above results appear to indicate that some melanoma cells, in common with a number of other malignancies, express CEA or CEA-like antigens on their surface. They also indicated that foetal antigens shown on melanoma cells in previous studies may, in part, be CEA-like antigens. However, foetal antigens other than CEA also appear to be expressed on melanoma cells, in that absorption on CEA of an antiserum known from previous studies to react with foetal antigens on melanoma cells, did not remove the activity of this antiserum against melanoma cells (Hersey *et al.*, 1976).

The precise identity of the antigens on the melanoma cell surface reacting with the CEA antiserum has not been defined in this study, and it is possible that they are molecules sharing antigenic determinants with CEA, and hence are CEA-like antigens. To some extent, description of CEA-reactive antigens as 'CEA' or 'CEA-like' appears arbitrary, in that CEA antigens from most sources appear to be heterogeneous (Coligan *et al.*, 1973; Harvey *et al.*, 1976). Studies to establish further the presence of CEA in melanoma cells by extraction procedures are in progress.

Before it can be accepted that CEA antigen is present on some melanoma cells, the possibility must be excluded that the reactivity of the anti-CEA serum may have been due to contaminating antibodies in the antiserum. This appears unlikely, in view of the rigorous method used in preparing the antigen and the extensive absorption procedures carried out on the antiserum raised against this antigen. Antigen was prepared from a metastasis of the colon in liver by established methods (Hughes, 1973, 1975) and gave a single precipitation line with the unabsorbed rabbit antiserum by immunoelectrophoresis and double diffusion in agar. These results conform to the criteria for CEA suggested by Terry *et al.* (1974). Absorption of the rabbit antiserum to CEA and the pregnancy antiserum, on an affinity column formed by coupling the CEA antigen to Con-A sepharose, removed the LDA activity to the melanoma cells. These results further indicated that the reactions were specific for CEA on the cell surface. Similar absorption procedures on rabbit anti-Chang serum did not remove the activity against the Chang cell, and absorption of several melanoma antisera did not remove the activity against melanoma cells which substantiated the specificity of the absorption procedure for CEA.

To our knowledge, there have been no previous descriptions of LDA activity against CEA antigens. Antibody-dependent killing of tumour cells is believed to be of possible importance in tumour rejection (Lamon *et al.*, 1972; O'Toole *et al.*, 1973; Hersey, 1973) and it would therefore appear feasible that CEA antigens may provide a target antigen for the immune defences against tumour growth. In the present studies however, we have not been able to detect antibodies to CEA antigens in several melanoma sera. Our failure to detect LDA may reflect an absence of an IgG antibody response in these patients or, alternatively, may result from absorption of the antibody by circulating tumour antigens. We have previously reported that absorption of LDA by melanoma antigens appears to be a common finding in patients with disseminated melanoma (Murray, McCarthy and Hersey, 1977).

Previous studies on antibody to CEA in the sera of tumour-bearing subjects have also shown a low incidence of antibody. Gold (1967) reported that IgM anti-CEA antibodies could be detected in approximately 70% of patients with non-metastatic digestive-system cancers, but subsequent study showed that many of these reactions were due to anti-A isoantibodies, and the true incidence of antibody to CEA may be much lower than this (Gold, Freedman and Gold, 1972). This also applied to the detection of CEA antibodies in sera from women during pregnancy, and the true incidence of CEA antibodies in pregnancy may be much lower than the figure of 70% initially reported by Gold (1967). In our present study, 5/15 women with pregnancies have shown reactivity to the melanoma target cell but we are unable to say without more extensive absorption studies whether this activity is due to anti-CEA antibodies in the sera.

Quite apart from the possible biological importance of CEA on melanoma cells, the present findings indicate that the application of assays for the detection of CEA antigens in melanoma sera may be of value in monitoring disease activity in melanoma patients, as has been described for a large variety of other tumours (Neville and Laurence, 1974). Studies to determine whether this is so are in progress.

We wish to thank Dr N. Hughes, Prince of Wales Hospital, Special Unit, New South Wales State Cancer Council, for supply of the CEA antigen and the rabbit antiserum to CEA and for his description of their preparation.

This work was supported by the Bill White Fund and the NSW State Cancer Council. We also wish to thank Mrs A. Edwards and Mrs E. Murray for their helpful assistance.

### REFERENCES

Coligan, J. E., Henkart, P. A., Todd, C. W. & Terry, W. D. (1973) Heterogeneity of the Carcinoembryonic Antigen. *Immunochemistry*, **10**, 591.

Gold, P. (1967) Circulating Antibodies against Carcinoembryonic Antigens of the Human Digestive System. *Cancer N.Y.*, **20**, 1663.

Gold, P. & Freedman, S. D. (1965) Specific Carcinoembryonic Antigens of the Human Digestive System. *J. exp. Med.*, **122**, 467.

Gold, J. M., Freedman, S. D. & Gold, P. (1972) Human Anti-CEA Antibodies Detected by Radioimmunoelectrophoresis. *Nature, New Biol.* **239**, 60.

Hall, R. R., Laurence, D. J. R., Darcy, D., Stevens, U., James, R., Roberts, S. & Neville, A. M. (1972) Carcinoembryonic Antigen CEA in the Urine of Patients with Urothelial Carcinoma. *Br. med. J.*, iii, 609.

Harvey, S. R., Girotra, R. N., Nemoto, J., Ciani, F. & Chu, T. M. (1976) Immunochemical Studies on Carcinoembryonic Antigen-reactive glycoproteins from Carcinomas of the Colon and Breast Separated by Concanavalin A Affinity Chromatography. *Cancer Res.*, **36**, 3486.

Hersey, P. (1973) A New Look at Antiserum Therapy of Leukaemia. *Nature, Lond.*, **244**, 22.

Hersey, P., Honeyman, M., Edwards, A., Adams, E. & McCarthy, W. H. (1976) Antigens on Melanoma Cells Detected by Leukocyte dependent Antibody Assays of Human Melanoma Antisera. *Int. J. Cancer*, **18**, 564.

Hughes, N. R. (1973) pH Dependent Changes in Composition of Carcinoembryonic Antigen. *Nature Lond.*, **243**, 523.

HUGHES, N. R. (1975) The Isolation and Characterization of Carcinoembryonic Antigen. M.Sc. Thesis, University of N.S.W.

JERRY, M. L., LEWIS, M. G., ROWDEN, G., SULLIVAN, A. K., PITZELE, R. & LAW, T. (1976) Fetal Antigens in Non-neoplastic Conditions. *Cancer Res.*, **36**, 3446.

LAMON, E. W., WIGZELL, H., KLEIN, E., ANDERSSON, B. & SKURZAK, H. M. (1973) The Lymphocyte Response to Primary Moloney Sarcoma Virus Tumors in BALB/c Mice. Definition of the Active Subpopulations at Different Times after Infection. *J. exp. Med.*, **137**, 1472.

LAURENCE, D. J. R., STEVENS, U., BETTELHEIM, R., DARCY, D., LEESE, C., TURBERVILLE, C., ALEXANDER, P., JOHNS, E. W. & NEVILLE, A. M. (1972) Evaluation of the Role of Plasma Carcinoembryonic Antigen (CEA) in the Diagnosis of Gastrointestinal, Mammary and Bronchial Carcinoma. *Br. med. J.*, iii, 605.

LEONG, P. L. S., SUTHERLAND, C. M. & KREMENTZ, E. T. (1977) Changes in Distribution of Human Malignant Melanoma Membrane Antigens in the Presence of Human Antibody by Immunofluorescence. *Cancer Res.*, **37**, 293.

MACHER, E., MULLER, C. H. R., SORG, G., GASSEN, A. & SORG, C. (1975) Evidence for Cross Reacting Membrane Associated Specific Melanoma Antigens as Detected by Immunofluorescence and Immune Adherence. *Behring Inst. Mitt.*, **56**, 86.

MOORE, T. L., ZUPCHIK, H. Z., MARION, N. & ZAMCHEK, N. (1971) Carcinoembryonic Antigen Assay in Cancer of the Colon and Pancreas and Other Digestive Tract Disorders. *Am. J. Dig. Dis.*, **16**, 1.

MURRAY, E., McCARTHY, W. H. & HERSEY, P. (1977) Blocking Factors against Leucocyte-dependent Melanoma Antibody in the Sera of Melanoma Patients. *Br. J. Cancer*, **36**, 000.

NERY, R., BULLMAN, H. & BARSOUM, A. L. (1973) Carcinoembryonic Antigens of Erythrocyte Membranes. *Nature* (*New Biol.*), **246**, 44.

NEVILLE, A. M., NERY, R., HALL, R. R., TURBERVILLE, C. & LAURENCE, D. J. R. (1973) Aspects of the Structure and Clinical Role of the Carcinoembryonic Antigen (CEA) and Related Macromolecules with Particular Reference to Urothelial Carcinoma. *Br. J. Cancer*, **28** (suppl.), 198.

NEVILLE, A. M. & LAURENCE, D. J. R. (1974) Report on the Workshop on the Carcinoembryonic Antigen (CEA). The Present Position and Proposals for Future Investigation. *Int. J. Cancer*, **14**, 1.

O'TOOLE, C., SJEJSKAL, V., PERLMANN, P. & KARLSSON, M. (1974) Lymphoid Cells Mediating Tumour-specific Cytotoxicity to Carcinoma of the Urinary Bladder. *J. exp. Med.*, **139**, 457.

TERRY, W. D., HEWKART, P. A., COLIGAN, J. E. & TODD, C. W. (1974) Carcinoembryonic Antigen: Characterization and Clinical Applications. *Transplant. Rev.*, **20**, 100.

VIZA, D., PHILLIPS, J. & TREJDOSIEWICZ, L. K. (1975) Cell Surface and Serum Melanoma Associated Antigens. *Behring Inst. Mitt.*, **56**, 83.